# Microbial Food Safety of Sous Vide Cooking Processes of Chicken and Eggs

**DOI:** 10.3390/foods13193187

**Published:** 2024-10-07

**Authors:** Miguel Romeo, Maria Lavilla, Félix Amárita

**Affiliations:** AZTI-BRTA, Food Research, 48160 Derio, Bizkaia, Spain; mromeo@azti.es (M.R.); famarita@azti.es (F.A.)

**Keywords:** food safety, microbial inactivation, sous vide (SV) cooking, *Campylobacter*, *Salmonella*, *Clostridium*

## Abstract

Sous vide cooking implies cooking foods, packed under vacuum conditions, at controlled temperatures (<80 °C). Although this method opens a new window of culinary possibilities, it also involves a series of risks, mainly microbiologically related, that must be assessed. The aim of this work was to evaluate the effectiveness of SV processes to inactivate three important foodborne pathogens (*Campylobacter*, *Salmonella*, and *Clostridium* spores) in chicken breast and eggs (omelet). For this purpose, two levels of inoculation (10^2^ and 10^6^ CFU/g), two different recipes, and two distinct treatments (with and without storage) for each food were studied. After treatments and storage, the corresponding microbiological counts were performed with standard methods. Average inactivation rates observed were 1.70, 4.82, and 4.34 log for *Clostridium* spores, *Campylobacter*, and *Salmonella*, respectively. No significant differences in microbial inactivation were perceived between the different recipes (food composition) or treatments, except for *Clostridium* spores, which showed a higher inactivation rate (2.30 log) when samples were stored. In general, preliminary results showed that, although appropriate levels of inactivation are reached for vegetative pathogenic cells, in some cases (spores in breast and *Salmonella* in eggs), the remaining microbiological risks should be considered and further studied, especially if long-term storage is planned.

## 1. Introduction

Stewing, by boiling food at temperatures close to 100 °C, or frying and baking, usually using higher temperatures, are the most common cooking procedures. However, nowadays, catering establishments are profusely developing new recipes that involve cooking at controlled temperature (45–80 °C): Sous vide (SV) cooking, as this method is commonly called, is a culinary technology proposed to enhance flavors and textures and improve the nutritional properties of food. By implementing this type of procedure, it is possible to keep aroma and get more juicy food as it is cooked in a sealed bag, enhancing nutritional quality and improving tenderness and sensory parameters [1,2,3]. To this purpose, SV combines vacuum and a precise temperature control, usually below 80 °C [4]. Accordingly, it also generally takes more time to accomplish the process than the standard procedures used in more traditional cooking. The recent increase in the use of these techniques, not only in the catering sector but also in the domestic sphere, is related to the appearance on the market of specific equipment to carry them out, such as immersion circulators, thermostatic baths, induction hobs with temperature control, slow cookers, steam ovens, or cooking robots. Another indispensable piece of equipment is the vacuum packing machine.

When considering the products most frequently cooked with this technique, poultry and eggs are widely consumed foods. However, due to their nature, these are also two foods very frequently implicated in foodborne toxicoinfections. More specifically, the two most reported zoonoses in humans were gastrointestinal diseases campylobacteriosis and salmonellosis, and the most frequently detected agents/foods pair in foodborne outbreaks with robust evidence, according to data provided by Member States, were *Salmonella* in “eggs and egg products” and *Campylobacter* in broiler meat and broiler meat products [5].

Also, taking into account that many of these products are frequently cooked under vacuum for improving organoleptic characteristics and that these foods may be stored after treatment for extended times [6], anaerobic temperature-resistant microorganisms, such as Clostridia (e.g., *Clostridium botulinum*), must be considered for safety evaluation. *Clostridium* thermoresistant spores germinate under favorable humidity, nutrient-present, and oxygen-absent conditions [7]. Since botulinic toxin is thermosensitive, botulism cases are related to ready-to-eat foods vacuum packaged. Also, *Clostridium perfringens* toxins in “other red meat or red meat mixtures and meat products” were the agent/food pair causing a high number of outbreak cases reported by European Union Member States [5]. To avoid these potential microbial issues, according to official international recommendations, food from animal origin must be completely cooked before consumption, reaching a temperature of at least 70 °C in the center of the product during more than two minutes for proper bacterial inactivation. Due to the increasing use of this technique, several sous vide cooking guidelines and works have been published [8,9,10], also recommending the proper combination of cooking temperatures and times for assuring food safety. However, microbiological quality depends on several parameters, such as the type of raw material, initial microbial counts, the presence of additives (e.g., marinate and brine composition), and thermal treatment factors.

Thus, the efficacy for inactivating foodborne pathogens when cooking with temperatures under 70 °C must be carefully checked [11]. Conventional predictive models for *Salmonella* foresee total inactivation at around 64 °C in less than 5 min, while for *Campylobacter* these temperatures could be lower, 55–60 °C [12,13,14]. This implies that recipes using higher temperatures could grant safe food. However, these models do not consider the entire culinary technique (controlled temperature combined with vacuum), and consequently, the fate of pathogenic microorganisms in SV products made with chicken meat and eggs is still not clearly known. In fact, some infections due to the misuse of these techniques have been reported [15]. The problem can be more evident when considering *Clostridium*, given its ability to form spores that predictably would resist these treatments.

Despite the importance of such studies, although the quality, structural, nutritional, and sensory aspects of foods have been deeply studied [16,17], limited publications can be found that focus on microbiological safety aspects of products that have been exclusively treated with sous vide procedures [1,9,11,18,19]. For instance, based on bibliography and assumptions of food thermal diffusivity, Baldwin [1] offered an estimation in D and Z values for *Listeria* and *Salmonella* inactivation, but no real inoculations or microbial counts were provided. Other works have studied microbial safety in sous vide processes, many of them focused on beef, seafood, pork, or lamb [19,20,21,22,23,24,25,26,27,28] and dealing with microorganisms such as *Listeria monocytogenes* [29]. Results found on this literature review are not comparable, beyond the high variability in microbial inactivation, since poultry products are subjected to more stringent inactivation values than red meats for safety [15,30]. Consequently, the microbiological quality of specific vacuum-packed poultry products is still a field to be explored.

According to this identified necessity, some recent works have begun research on the microbial safety of SV cooking in chicken breast and similar products, such as turkey breast or chicken ham [31,32,33,34]. These works mainly dealt with the natural flora (total plate count and presence of relevant species) of treated products [32,33,34]. Only Hasani et al. [31] performed inactivation experiments with one strain of *Enterococcus faecalis*, but other microorganisms of main concern, such as *Campylobacter*, have been barely studied [35].

Likewise, there are very few original studies focused on the microbiological quality of eggs treated by SV. Although some references have been found on the safety of shell eggs [9], whole egg omelets subjected to SV processing still lack specific studies considering the inactivation of *Salmonella*.

As perceived, limited data have been previously provided about poultry products cooked under SV from a microbial food safety point of view with inoculated samples, and more studies are needed to understand and bring this promising culinary tool to a safer level [11]. Thus, the purpose of this work is to evaluate the effectiveness of controlled-temperature processes (≤75 °C) over long periods of time under vacuum conditions to inactivate three significant foodborne pathogens (*Salmonella*, *Campylobacter*, and anaerobic spores) in frequently sous vide-cooked foods (chicken breast and eggs). Our intention was to study differences among some specific treatments (considering also storage), looking for potential conditions that are likely to be used but may cause relevant microbial threats. Finally, this work aims to acquire preliminary new data about the ability of significant pathogens to survive SV processing and their ability to subsequently grow during prolonged periods, providing specific inactivation rates under limited conditions to advance knowledge about the risk associated with the consumption of these products.

## 2. Materials and Methods

### 2.1. Microbial Strains

The strains used in this study are related in Table 1. *Clostridium sporogenes* was selected as a usual surrogate of *Clostridum botulinum* spores in terms of thermal resistance [36], but with fewer handling hazards. As said before, *Salmonella* and *Campylobacter* were selected as representative of the two more frequent microorganisms causing foodborne diseases [5], mainly associated with eggs and chicken foods.

For trials, bacteria from cryovials (stored at −80 °C in 20% glycerol) were initially transferred to a tube containing soybean casein digest, brain heart infusion, and reinforced clostridial broths for *Salmonella*, *Campylobacter*, and *Clostridium* recovery, respectively. *Salmonella* was incubated at 37 °C for 24 h. *Campylobacter* was incubated in microaerobic conditions (GasPak™ EZ Campy, BD Difco, Franklin Lakes, NJ, USA) at 41.5 °C for 48 h, and *Clostridium sporogenes* was incubated under anaerobic conditions (AnaeroGen™, Oxoid-Thermo Scientific, Hampshire, UK) at 37 °C for 48 h.

Subsequently, liquid cultures were spread in agar plates containing XLD or RapidCampy agar for *Salmonella* or *Campylobacter*, respectively. The preparation of the inoculation suspensions was carried out by resuspending the microorganisms in McFarland solution to a final concentration of ≈2.5 × 10^8^ CFU/mL.

For *C. sporogenes*, sporulation was carried out by a dialysis-sac culture device as described before [37] with slight modifications. Briefly, a volume of 20 mL from a 20 h grown culture of the strain in reinforced clostridium medium (RCM, Scharlau) was inoculated into a MWCO 12–14,000 cellulose dialysis membrane immersed into tryptone–glucose–yeast extract (TGY) sporulation medium [38] and incubated under anaerobic conditions at 37 °C for at least 48 h. The suspension obtained was centrifuged at 4000× *g* for 15 min at 4 °C and washed three times with sterile distilled water. After cleaning of the suspension, absence of vegetative cells was checked by observation of the suspension under phase-contrast microscopy (PCM), and spore numbers were measured by direct culture of 10-fold dilutions in RCA (reinforced clostridial agar) in anaerobic conditions. Once assessed, spore concentration was adjusted to 2.5 × 10^8^ spores/mL with NaCl 0.85% for inoculation.

### 2.2. Foods Preparation

Egg and chicken breast were selected as model foods for this study based on the frequency of utilization for SV cooking recipes, the facility to standardize the units of study, as well as their association with foodborne outbreaks related to *Salmonella* and *Campylobacter*, respectively. Raw filleted chicken breasts and liquid pasteurized eggs were purchased from a local supermarket. Raw products were checked for the absence of natural flora that could interfere with the results prior to inoculation with the corresponding microbial methods described below.

Two recipes for each food were selected in order to study other parameters potentially influencing the inactivation, such as the presence of condiments (spices) (Table 2). Spiced breast samples (25 g) were initially submerged in a 10% NaCl brine for 1 h. Then, they were impregnated on their surface with a season composed of garlic (2 g), black pepper (0.5 g), dill (0.5 g), and basil (0.5 g) in olive oil (450 mL) by immersion of pieces and gentle manual homogenization for 10 min. Eggs, in both natural and spiced preparations, were prepared by adding 0.4% NaCl. For spiced egg recipes, liquid egg was also added with 20% chopped onion, 10% olive oil, and 1% dill.

Contamination was carried out at two levels: ≈10^2^ CFU/g and ≈10^6^ CFU/g. The purpose of the two levels of contamination was to study if the treatments were enough to inactivate all the microorganisms in foods (usually present in low numbers, using as model concentration 1 × 10^2^ CFU/g) and, on the other hand, study the real ability of inactivation (number of log reduction) of the process (inoculation of 10^6^ CFU/g). For raw filleted chicken breasts, 25 g samples were sorted for similar thickness and surface appearance. Then, 100 µL of the corresponding bacterial suspension dilution to reach the desired concentration was spread homogeneously on the surface with a Drigalski spatula. Liquid egg samples (25 g) were inoculated by gentle manual mixing of the product with the corresponding microbial suspension for uniform contamination.

### 2.3. Sous Vide Cooking

Two different sous vide treatments were selected for each food product (Table 2), based on recommended temperature/time combinations from previous publications [1,39,40,41], and the real frequency of use, inferred from a previous survey to 10 collaborator restaurants regularly using this technique: (1) a “regular” treatment (the one most frequently used in referred restaurants: 65 °C/75 min for chicken breast or 75 °C/10 min for egg) and (2) a “critical” treatment (theoretical temperature/time combination with plausible risk from a microbiological point of view according to the bibliographic data consulted), combined with an extended storage at 6 °C (including the possibility of a not-immediate consumption).

Samples were vacuum packed (0.9 mbar) in 120-micron PA/PE bags (165 × 200 mm) using a tabletop vacuum machine (C200, Multivac, Wolfertschwenden, Germany) and then totally submerged in a thermostatic water bath (Optima GD120, Grant Instruments, Royston, UK) at the set temperature for the corresponding time. A set of samples remained untreated for inoculation control. After sous vide thermal processing, samples were immediately cooled down in iced water, and those with no storage period were directly analyzed. The rest of the samples were stored at 6 °C for 14 days (breast) or 7 days (egg).

### 2.4. Microbial Counts and Investigations

Samples were initially diluted 1/10 with 0.1% buffered peptone water (BPW, Pronadisa, Laboratorios CONDA, Madrid, Spain) in sterile bags in a Stomacher 400 (Seward Ltd., West Sussex, UK) for one minute. Then, additional 10-fold dilutions were prepared in BPW when necessary.

For *Campylobacter* enumeration, 100 µL of the corresponding dilution of the chicken breasts was spread onto RAPID’Campylobacter (Bio-Rad, Hercules, CA, USA) agar plates and incubated at 41.5 °C for 48 h under microaerophilic conditions (GasPak™ EZ Campy, BD Difco, Franklin Lakes, NJ, USA).

For *Salmonella* enumeration, 100 µL of the corresponding dilution of omelets was spread onto xylose–lysine–deoxycholate (XLD) agar plates (bioMerieux, Marcy-l’Étoile, France) and incubated at 37 °C for 24 h in aerobic conditions.

In addition to agar counts procedures, for the detection of *Salmonella* and *Campylobacter*, standardized procedures based on real-time PCR (iQ-check Kit, Bio-Rad, Hercules, CA, USA) were also followed, following manufacturer instructions.

For *Clostridium* enumeration, dilutions from chicken breast were inoculated in RCA (reinforced clostridial agar) plates and incubated for 48 h at 37 °C in anaerobiosis (AnaeroGen™, Oxoid Thermo Scientific, Hampshire, UK).

In non-inoculated chicken breast samples, natural flora was analyzed by total aerobic plate counts, yeast and molds, *Enterobacteriaceae*, lactic acid bacteria (LAB), and *Pseudomonas* counts: total aerobic mesophilic plate count was performed by pouring 1 mL of the corresponding dilution on Petrifilm AC (3M, Madrid, Spain) and incubating at 30 °C for 48 h. For the plate count of *Enterobacteriaceae*, 1 mL of the corresponding dilution was tested on Petrifilm EB as specified by the manufacturer and incubated at 37 °C for 24 h. After incubation, colonies identified as *Enterobacteriaceae* were counted. Products were also analyzed for lactic acid bacteria (LAB) and yeast and mold concentrations. For this purpose, 1 mL of the corresponding 10-fold dilution of the homogenate was spread on the surface of Petrifilm LAB or Petrifilm RYM and incubated for 48 h at 37 °C or 25 °C, respectively. Finally, *Pseudomonas* were counted in Pseudomonas agar base (Oxoid, UK) supplemented with CFC (cetrimide/fucidin/cephalosporin) Pseudomonas selective supplement (Oxoid, UK) after incubation at 25 °C for 48 h.

All the microbiological counts were transformed into logarithmic units, as the log_10_ of colony-forming units per gram of product (CFU/g).

### 2.5. Statistical Analysis

All experiments were conducted in triplicate with three individually contaminated samples per condition and product in each experiment (*n* = 9). The corresponding inactivation rate for each microorganism and product was calculated as the number of spores and/or cells in treated samples compared to the total number of spores and/or cells in control (untreated) samples. Statistical analysis was performed on the means of the corresponding microbial counts with Statgraphics Centurion Statistical Software (Version 16.2.04, StatPoint Technologies, Inc., Warrenton, VA, USA). To determine differences in inactivation rates of each microorganism among the different tested conditions, looking for potential conditions that are likely to be used but may cause relevant microbial threats, data were analyzed using the analysis of variance (ANOVA) at the level of significance *p* = 0.05.

## 3. Results and Discussion

In order to estimate the safety of SV processes, knowing the inactivation rates of the most significant pathogens in each food seems like an interesting approach. The selection of studied microorganisms in challenge testing for microbial inactivation studies should be a result of an assessment of the risk for food contamination and of the intrinsic and extrinsic characteristics of the food to support microbial growth [42]. Consequently, and according to recent surveillance data about the incidence of foodborne diseases and implied foods, the studied microorganisms were chosen for this study [5]: *Campylobacter* for chicken breast and *Salmonella* for egg omelet. *Clostridium sporogenes* spores were selected as an extra indicator of microbial risk considering pH, controlled thermal treatment, and vacuum package of chicken breast samples.

### 3.1. Inactivation of Clostridum Spores in Chicken Breast

**Figure 1:**
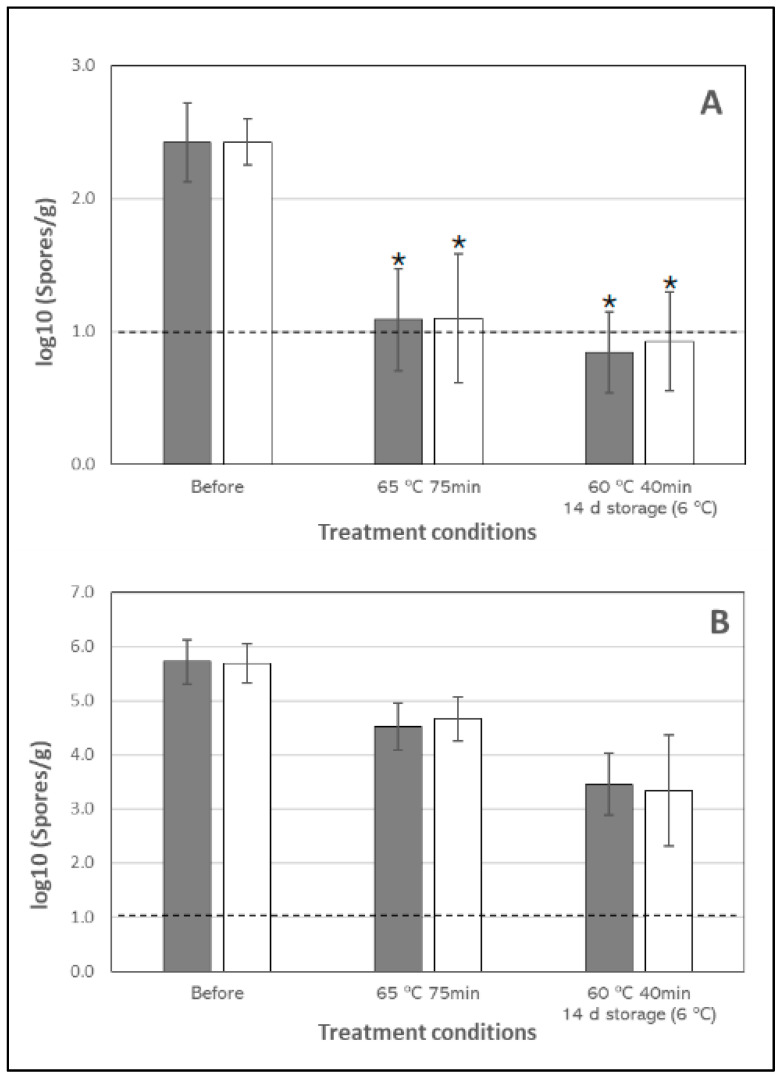
*Clostridum sporogenes* counts (log_10_ spores/g) in natural (■) or spiced (□) chicken breast before and after selected treatments for the two levels of inoculation studied: (**A**) 10^2^ spores/g; and (**B**) 10^6^ spores/g. Horizonal dotted line indicates the limit of detection of the method used, and asterisk indicates estimated counts. * Estimated value (some counts were under the limit of detection). Vertical error bars indicate standard deviation (*n* = 9).

The results obtained indicate a slight reduction in *Clostridium* spore loads (Figure 1, Table 3). Medium inactivation rates were calculated in samples inoculated at 10^6^ spores/g to be 1.02 log and 1.19 log for natural and spiced breasts, respectively, treated at 65 °C for 75 min. When the treatment at 60 °C for 40 min (after 14 days of storage) was considered, we found higher inactivation rates: 2.26 log and 2.34 log for natural and spiced breast, respectively. Viewing these results, no significant differences were detected between the seasoned and natural products in any of the studied conditions (Table 3).

Spores are resistance forms, and consequently, they are resistant to intense thermal treatments. However, slight inactivation rates (up to 3 logs) were observed despite the used temperatures, below 80 °C, probably due to the long times used in selected treatments. Due to the known heat resistance of spores, it is hard to find previous studies dealing with spore inactivation at temperatures lower than 90 °C, highlighting the importance of this kind of research on sous vide processes. Considering traditional thermal-inactivation models, for instance, Byrne et al. [43] achieved an inactivation rate of 6 log at 70 °C. Similarly, El Kadri et al. [44] assessed that high inactivation rates (at least 6 log) may be achieved at 55 °C. However, these works were performed with vegetative cells, and results are not equivalent. For *Clostridium* spores, D-values from 2.2 min (100 °C) to 34.2 min (90 °C) were previously obtained [43], so it is assumable that at 70–75 °C for much longer times, as used in sous vide cooking, may achieve observed minor inactivation that indicates that these controlled-temperature treatments may not be enough to reduce this microbiological hazard [11,45]. Also, it must be considered that traditional thermal inactivation models (based on linear kinetics) may not be directly applicable to this range of temperatures [11]. In previous works dealing with SV processes, *Clostridium perfringens* spores were not detected in turkey cutlet after sous vide (65 °C for 40 min and cold storage), but only natural microbial contamination was taken into account, and initial counts (prior to treatment), presumably low, are not provided to calculate real inactivation rates [46].

In samples inoculated at a lower concentration of spores (10^2^ spores/g), inactivation rates could only be estimated. The results showed that, although in some samples microbial counts were under the limit of detection of the method used (Figure 1), in most cases, these treatments are not sufficient to effectively inactivate the spores if they are present in foods, even at low concentrations. Consequently, these microorganisms are likely to remain viable after SV treatments. However, our results also showed that spores were not able to germinate and grow at the studied refrigeration temperatures (6 °C). Contrarily, although no differences were detected between the spiced and natural products, significant differences were detected between the regular (65 °C/75 min) and the critical (60 °C/40 min/storage) treatment: as shown by the results, we observed a higher inactivation rate after 14-day storage, although lower temperature and time of exposure were used for thermal treatment. Assuming that pouches were properly sealed and that a softer thermal treatment caused lower inactivation of spores, the possible explanation for a higher inactivation should be focused on storage conditions and the method of determining the spore counts. It has been shown that SV vacuum packs may contain some residual oxygen. However, apparently, these levels would not be enough to inhibit the growth of *Clostridium* spores [9,47]. Thus, another explanation would be related to the potential germination and recovery of the remaining spores. Beyond thermal inactivation, controlled temperature may also activate spore germination and growth during subsequent storage [48], which is the most serious threat to safety concerning spore-forming pathogens [11,49]. However, although pH and atmosphere conditions could be favorable, the fast-cooling step and the cold temperatures used in storage would prevent the growth at studied refrigeration temperatures (6 °C) in the studied storage times, which are relatively short [18,50,51]. Additionally, the hydration of the core during spore germination that may occur during SV treatment allows microbial metabolism to restart but concurrently leads to a loss of resistance [50], reflected in a loss of viability and lower microbial counts due to the method used (cultivable microorganisms). These results enhance the known guidance that quick cooling and proper storage conditions must be carefully observed in order to guarantee microbial safety, apart from selecting the appropriate cooking temperature and time.

### 3.2. Inactivation of Campylobacter in Chicken Breast

As values indicated in Table 3 denote, both treatments were able to inactivate *Campylobacter* species, reaching, in all cases, plate counts under the limit of detection, also in the samples contaminated with higher concentrations (10^6^ CFU/g). PCR analysis confirmed these results, since this microorganism was not detected in any treated sample. Thus, the average reduction rate of this microorganism was higher than 5.82 logarithmic units for both treatments in this study. No significant differences were observed between natural and seasoned chicken, nor between treatments.

Contrarily to spores, treatments were able to effectively inactivate *Campylobacter* spp. These results are in agreement with previous model predictions for this bacterium with conventional thermal treatments [13,14]. Considering sous vide-treated samples, similar results were also obtained in chicken meat [35]: with analogous levels of inoculation (10^6^ CFU/g), when samples were cooked at both 60 and 65 °C, all samples were negative after 60 min of treatment. However, it is remarkable that, after 30 min of treatments, observed inactivation rates were only 0.6 and 1 log at 60 and 65 °C, respectively. These differences rely on the different sample sizes studied (25 g vs. 300 g). This highlights the importance of reaching the desired temperatures at the center of the cooked piece. Since change in color or texture, the typical checkpoint in restaurants and households, may occur in chicken under 60 °C [35,52,53,54], this does not assure the appropriate inactivation of pathogens. Consequently, it becomes critical to perform further microbial studies with the objective of controlling factual microbial inactivation in different circumstances.

### 3.3. Effect on Natural Flora of Chicken Breast

As observed in Table 4, chicken breasts before the treatments presented high counts of total aerobic microorganisms, ranging between 4.17 and 5.20 log_10_(CFU/g). Within the specific controlled groups, *Pseudomonas* was predominant, exhibiting levels of about 10^4^ CFU/g. Considering lactic acid bacteria, molds and yeasts, and enterobacteria, concentrations ranged between 10^2^ and 10^3^ CFU/g.

For all considered microbial groups, post-treatment reductions in counts were observed (Table 4), although it was most evident in total aerobic counts, where average inactivation rates were by 3.39 orders of magnitude. In this case, small but significant differences were observed between treatments and the kind of product. Globally, the treatment at 65 °C for 75 min caused the greatest average reduction, 3.57 log, while less intense treatments and storage resulted in lower inactivation rates. Similarly, the inactivation values were higher in natural samples than in spiced products.

In this case, less than 5 log cycles of inactivation were achieved. Results found in previous works are very variable [49]. However, most of previous works report inactivation rates ranging from 2–3 log CFU/g in total mesophilic microorganisms [25,32,52], in agreement with our results. Also, we report lower inactivation rates with a less intense treatment and subsequent storage. Although non-detectable viable counts are detected in most samples immediately after treatment, after long-term refrigerated storage (7–14 days), samples presented higher counts of total mesophilic and psychrophilic bacteria [26,34], confirming the impact of storage in culinary samples. Also, we have confirmed the importance of performing microbial studies in different conditions, since the composition of the media may impact the inactivation of some microorganisms, as seen in this work. Samples with higher fat content (olive oil in condiment) or spices also showed lower inactivation rates in previous works [55,56,57]. Consequently, although studied thermal treatments have been confirmed to achieve inactivation rates of at least 5 log of the inoculated pathogenic microorganisms, chicken breast treated by SV could be consequently still exposed to microbial spoilage [49], which should be considered especially in products intended for long storage periods.

For the rest of the microorganisms, both treatments induced a decrease in overall counts below the limits of detection of the methods used. Due to the lower initial counts, inactivation rates could not be properly calculated, and no significant differences could be evidenced among treatments or products to suggest that the variables can have a significant effect on natural flora reduction. However, previous studies have also observed similar inactivation rates in SV-treated products, including chicken breast, to undetectable levels in *Enterobacteriaceae* [25,32], *Pseudomonas* [26], or yeast counts [26], confirming our results.

### 3.4. Inactivation of Salmonella in Egg (Omelet)

The results obtained from the study on eggs contaminated with *S. enterica* (Table 5) showed acceptable thermal inactivation when considering microbial counts. However, although the plate counts in treated samples were in all cases under the limit of detection of the method used (less than 10 CFU/g), *Salmonella* was still detected by PCR in all the treated samples inoculated at a theoretical concentration of 10^6^ CFU/g. Thus, considering the real initial concentration (5.34 logs on average), the reduction in *Salmonella* contamination was estimated to be 4.34 logs, with no significant differences between seasoned and natural samples nor between treatments.

In the case of eggs, studied treatments are not able to fully eliminate *Salmonella* since PCR analysis detected possible viable cells. Previously published inactivation models (traditional thermal inactivation) forecast that similar reduction rates may be obtained in liquid whole eggs with considerably lower temperatures (58–60 °C) for 5–12 min [12,58]. Also, *Salmonella* was not detected in sous vide-treated meats [25,34,46,52]. However, in these works, natural initial counts of *Salmonella* were low or negative (absence of *Salmonella* in 25 g of food) in untreated samples. Contrarily, our results suggest that special conditions of eggs for SV treatments could reduce *Salmonella* inactivation. As a consequence, although these treatments would be considerably close to accomplishing a stablished acceptability limit to be considered safe (inactivation rates of at least 5 log of inoculated microorganisms), and the initial level of contamination (~2 × 10^5^ CFU/g) is unlikely in actual samples, our results notice once again the importance of individually checking the safety on sous vide processes and the importance of appropriate refrigerated storage if consumption of SVPL-treated eggs is not immediate.

## 4. Conclusions

As deduced from this work, this kind of study is needed to fully understand the inactivation of microorganisms and subsequent microbial safety of treated products under the specific conditions of sous vide (SV) cooking.

In general, it can be concluded that SV can be used as preservation technology, with confirmed effects against the studied vegetative microorganisms. However, when considering specific microorganisms, the investigation suggested that treated products may be considered still exposed to a potential microbiological risk since inactivation is not completely effective. Thus, the study and selection of proper treatment conditions (temperature/time combination) depending on the specific risks of each group of food is critical to avoid hazards. These results must be specially taken into account when treated samples are intended for subsequent storage and not immediate consumption.

The results presented in this work provide preliminary and limited data but highlight the importance of scrutiny for relevant microbial risks and checking for potential conditions that are likely to be used but may cause relevant microbial threats, especially because the microbiological quality depends on numerous parameters, such as the composition of raw material, initial microbial counts, and the thermal treatment factors applied, as seen in this work. Considering the high variability in foods and different processes performed by restaurants, further studies on different food compositions, different microorganisms’ species/strains, and mechanisms behind the observed results (especially with spore-forming species) should be performed to complete the overall assessment of SV microbial safety.

## Figures and Tables

**Table 1:** Bacterial strains that were used in this study.

Species	Strain Designation	Source (Code)
*Salmonella enterica* *Subsp. enterica*	Serotype: 4,5,12:i:1,2; Typhimurium	CECT 4156
*Campylobacter coli*	serovar 4	CECT 8205
*Campylobacter jejuni*	serovar 2	CECT 8170
*Clostridium sporogenes*	McClung 2004	CECT 892

**Table 2:** Summary of the studied conditions.

Food	Recipe	Initial Counts (CFU/mL)	Microorganism	Treatment	Storage (6 °C)
Chicken breast	Natural chicken breast	Non-inoculated	Natural flora	65 °C, 75 min	-
				60 °C, 40 min	14 d
		~10^2^	*Campylobacter* *Clostridium*	65 °C, 75 min	-
				60 °C, 40 min	14 d
		~10^6^	*Campylobacter* *Clostridium*	65 °C, 75 min	-
				60 °C, 40 min	14 d
	Spiced chicken breast	Non-inoculated	Natural flora	65 °C, 75 min	-
				60 °C, 40 min	14 d
		~10^2^	*Campylobacter* *Clostridium*	65 °C, 75 min	-
				60 °C, 40 min	14 d
		~10^6^	*Campylobacter* *Clostridium*	65 °C, 75 min	-
				60 °C, 40 min	14 d
Liquid egg	Natural omelet	~10^2^	*Salmonella*	75 °C, 10 min	-
				70 °C, 5 min	7 d
		~10^6^	*Salmonella*	75 °C, 10 min	-
				70 °C, 5 min	7 d
	Spiced omelet	~10^2^	*Salmonella*	75 °C, 10 min	-
				70 °C, 5 min	7 d
		~10^6^	*Salmonella*	75 °C, 10 min	-
				70 °C, 5 min	7 d

**Table 3:** Average inactivation rates (in log CFU/g) in chicken breast (*n* = 9) at selected treatments.

Parameter	Additives (Spices)	Inoculated 10^2^		Inoculated 10^6^	
		65 °C 75 min	60 °C 40 min 14 D	65 °C 75 min	60 °C 40 min 14 D
*Clostridium* spores	Natural	>1.23 ± 0.5 *^,^**	>1.36 ± 0.38 *^,^**	1.19 a ± 0.17	2.26 b ± 0.23
	Spiced	>1.18 ± 0.37 *^,^**	>1.32 ± 0.29 *^,^**	1.02 a ± 0.35	2.34 ab ± 1.05
*Campylobacter* spp.	Natural	>2.19 ± 0.18 ***	>2.19 ± 0.18 ***	>5.87 ± 0.21 ***	>5.87 ± 0.21 ***
	Spiced	>2.07 ± 0.44 ***	>2.07 ± 0.44 ***	>5.77 ± 0.16 ***	>5.77 ± 0.16 ***

* Counts in treated samples under the limit of detection, ** Estimated counts in treated samples, *** not detected by PCR in treated samples. Different letters indicate groups presenting significant differences (*p* < 0.05).

**Table 4:** Inactivation rates (log) of natural flora in chicken breast at selected SV treatments.

Parameter	Additives (Spices)	Initial Counts (log_10_ CFU/g)	Inactivation Rates	
			65 °C 75 min INST	60 °C 40 min 14 D
Total aerobic counts	Natural	5.06 ± 0.09	4.06 a ± 0.09	3.46 b ± 0.09
	Spiced	4.73 ± 0.52	3.08 bc ± 0.51	2.94 c ± 0.33
*Enterobacteriaceae*	Natural	2.84 a ± 0.03	>1.84 ±0.03 *	>1.84 ± 0.03 *
	Spiced	2.00 b ± 0.00 *	>1.00 ± 0.00 *	>1.00 ± 0.00 *
*Pseudomonas*	Natural	4.06 ± 0.29	>2.06 ± 0.29 *	>2.06 ± 0.29 *
	Spiced	3.40 ± 0.35	>1.40 ± 0.35 *	>1.40 ± 0.35 *
LAB	Natural	3.28 ± 0.31	>2.28 ± 0.31 *	>2.28 ± 0.31 *
	Spiced	2.95 ± 0.60	>1.95 ± 0.60 *	>1.95 ± 0.60 *
Yeast and molds	Natural	2.00 ±0.00 *	>1.00 ± 0.00 *	>1.00 ± 0.00 *
	Spiced	2.40 ± 0.35	>1.40 ± 0.35 *	>1.40 ± 0.35 *

* Counts in treated samples under the limit of detection. Different letters indicate groups presenting significant differences (*p* < 0.05). LAB: lactic acid bacteria.

**Table 5:** *Salmonella* inactivation rates (log) in egg.

		Inoculated 10^2^		Inoculated 10^6^	
Product	Initial Concentration	75 °C 10 min	70 °C 5 min 7 D	75 °C 10 min	70 °C 5 min 7 D
Natural omelet	5.33 ± 0.3	>1.76 ± 0.19 *^,^**	>1.76 ± 0.19 *^,^**	4.33 ± 0.3 *^,^***	4.33 ± 0.3 *^,^***
Spiced omelet	5.35 ± 0.18	>1.84 ± 0.15 *^,^**	>1.84 ± 0.15 *^,^**	4.35 ± 0.18 *^,^***	4.35 ± 0.18 *^,^***

* Counts in treated samples under the limit of detection, ** not detected by PCR in treated samples. *** PCR positive in treated samples.

## Data Availability

The data presented in this study are available on request from the the main author (famarita@azti.es) because of privacy restrictions.
